# Predictive In Vitro Diagnostic Screening of Strontium-Enriched Biodegradable Mg–Ca Alloys for Emerging Dental Applications

**DOI:** 10.3390/diagnostics16071060

**Published:** 2026-04-01

**Authors:** Kamel Earar, Ciprian Adrian Dinu, Marius Valeriu Hînganu, Gabriela Leață, Corneliu Munteanu, Cristian Constantin Budacu

**Affiliations:** 1 Faculty of Medicine and Pharmacy, Dunarea de Jos University of Medicine and Pharmacy, 800008 Galati, Romania; kamel.earar@ugal.ro (K.E.); gabrielaleata@yahoo.com (G.L.); 2 Faculty of Medicine, Grigore T. Popa University of Medicine and Pharmacy, 700115 Iasi, Romania; cristian.budacu@umfiasi.ro; 3 Mechanical Engineering Faculty, “Gheorghe Asachi” Technical University of Iasi, 700050 Iasi, Romania; corneliu.munteanu@academic.tuiasi.ro; 4 Technical Sciences Academy of Romania, 030167 Bucharest, Romania

**Keywords:** in vitro diagnostics, predictive screening, biodegradable magnesium alloys, strontium, osteoblast-like cells, dental biomaterials, extract testing, digital workflow, ISO 10993, cytocompatibility profiling

## Abstract

**Background**: Biodegradable magnesium-based alloys are increasingly explored as emerging biomaterials for dental and maxillofacial applications due to their osteoconductive properties and potential to reduce long-term implant-related complications. However, early-stage evaluation requires predictive diagnostic screening methods capable of assessing cytocompatibility and cellular response under clinically relevant extract conditions. **Objectives**: In this study, Mg–0.5Ca alloys modified with increasing strontium concentrations (0.5–3 wt.%) were investigated through an in vitro diagnostic framework using MG-63 osteoblast-like cells. **Methods**: Cell viability was quantitatively assessed via MTT assays after 24 and 72 h of exposure, while fluorescence-based live-cell imaging provided complementary morphological insights. **Results**: demonstrated a composition-associated cytocompatibility profile, with Sr-enriched compositions showing improved cellular metabolic activity and adhesion patterns compared to lower-Sr compositions. **Conclusions**: These findings support the role of strontium as a functional alloying element and highlight the importance of standardized diagnostic screening workflows for emerging dental biomaterials. Overall, this study proposes a simplified predictive platform for early biocompatibility diagnostics, contributing to the integration of biomaterial evaluation into future digitalized dental regeneration workflows.

## 1. Introduction

The development of biodegradable biomaterials has transformed the way temporary implants are conceived in bone repair and tissue engineering. Unlike permanent metallic devices, biodegradable materials are designed to provide mechanical support only for the time required for healing, after which they gradually resorb and are replaced by newly formed tissue, thereby eliminating the need for secondary removal surgery and reducing long-term complications. Among the three main families of resorbable biomaterials—polymers, ceramics, and metals—biodegradable metals have attracted particular attention because they combine high load-bearing capacity with bioactivity and a degradation profile that can, in principle, be tuned to match the pace of tissue regeneration. Recent reviews consistently highlight magnesium-, zinc-, and iron-based alloys as the most promising candidates for next-generation temporary implants, especially in orthopaedic and maxillofacial applications [1,2,3,4].

Magnesium-based alloys occupy a central place in this landscape due to their elastic modulus and density being closer to those of cortical bone than conventional implant metals such as titanium or stainless steel, which helps to mitigate stress shielding and promote physiological load transfer [2,5]. In addition, magnesium is an essential ion involved in numerous enzymatic and signalling pathways relevant to bone metabolism. However, the clinical translation of Mg-based implants is still limited by their high corrosion rates in physiological environments, hydrogen gas evolution, and local alkalinisation, which can compromise early mechanical stability and cellular responses [3,6,7].

As a result, current research focuses on tailoring alloy composition and surface state in order to reconcile mechanical integrity, controlled biodegradation, and biological performance.

Among the different alloying strategies proposed, the incorporation of calcium and strontium into Mg matrices has emerged as particularly attractive for skeletal applications. Both elements are naturally present in bone and play active roles in bone remodelling and mineralisation. Calcium is essential for hydroxyapatite formation and contributes to improved biocompatibility and microstructural refinement when used as an alloying element in Mg–Ca systems [8,9]. Strontium, in turn, is known to exert a dual action on bone cells, stimulating osteoblastic differentiation and matrix synthesis while concurrently attenuating osteoclast-mediated resorption, a combination that has led to its use in systemic therapies for osteoporosis and to the design of strontium-doped bioactive ceramics, glasses, and coatings [10,11,12]. Higher Sr-containing alloys were associated with enhanced viability responses, although direct Sr^2+^ quantification was not performed.

More recently, there has been growing interest in exploiting Sr doping directly within metallic substrates or hybrid scaffolds to couple mechanical support with osteoinductive ion release [13,14].

Within this context, Mg–Ca–Sr alloys represent a logical evolution of Mg–Ca systems, aiming to integrate the structural and biological functions of all three elements. Experimental and review studies published in the last few years indicate that judicious addition of Sr to Mg–Ca alloys can refine the microstructure, modify corrosion behaviour, and enhance osteogenic activity, although excessive Sr contents may promote galvanic corrosion through the formation of intermetallic phases with higher nobility [15,16].

In vitro and in vivo evaluations of Mg–Ca–Sr-based materials, including recent work on cast Mg–0.5Ca–xSr alloys, have shown that these systems can achieve acceptable degradation rates, favourable tissue responses, and promising bone integration when composition and processing are carefully controlled [17,18].

At the cellular level, understanding how osteoblasts and osteoblast-like cells respond to degradation products released by Mg–Ca–Sr alloys is crucial for their rational design and safe implementation. Many in vitro studies on Mg-based biomaterials have focused on cell viability, proliferation, and differentiation in the presence of alloy extracts or direct-contact cultures, using cell types such as human bone marrow mesenchymal stem cells, primary osteoblasts, or osteoblast-like lines. These investigations demonstrate that moderately elevated concentrations of Mg^2+^, Ca^2+^, and Sr^2+^ can support or even stimulate osteogenic activity, while excessively rapid corrosion and associated changes in osmolality or pH are detrimental [4].

Nonetheless, systematic comparisons across a controlled series of Mg–Ca–Sr compositions, particularly using a widely employed osteoblast-like model such as MG-63, remain relatively scarce. In addition, the combined analysis of metabolic activity and morphological adaptation of osteoblast-like cells in response to graded alloy extracts has not yet been fully clarified for this specific class of biodegradable alloys.

Building on this background, the present work investigates the in vitro cytocompatibility and morphological response of osteoblast-like MG-63 cells to degradation extracts derived from a series of cast Mg–0.5Ca–xSr (x = 0.5, 1, 1.5, 2, 3 wt.%) biodegradable alloys. These alloys have previously been characterised in terms of microstructure, corrosion behaviour, and in vivo biocompatibility, demonstrating their potential as candidates for temporary metallic implants in dental and orthopaedic applications [17,18].

Here, we focus specifically on the cellular response, quantifying metabolic activity by MTT assay after 24 h and 72 h exposure to graded extract dilutions and correlating these findings with fluorescence-based assessment of cell morphology. By linking alloy composition, extract concentration, and osteoblast-like behaviour, this study aims to contribute to a more nuanced understanding of the design space for Mg–Ca–Sr biodegradable alloys and to provide experimental support for their further development in bone tissue engineering.

While extensive research has addressed the microstructural, electrochemical, and mechanical characterization of magnesium-based biodegradable alloys, fewer studies have focused specifically on the cellular response to degradation extracts across controlled compositional gradients. In particular, systematic investigations correlating strontium content with osteoblast-like cell metabolic activity and morphology remain limited.

Early-stage evaluation of novel dental biomaterials increasingly relies on predictive in vitro diagnostic assays, which provide rapid and standardized insights into cytocompatibility prior to complex animal or clinical validation. Such diagnostic screening platforms are essential for identifying optimal alloy compositions, minimizing biological risks, and accelerating translational pathways in regenerative dentistry.

Furthermore, the development of emerging biomaterials is progressively connected to digital dentistry workflows, where CAD/CAM-driven implant design and personalized regenerative solutions require parallel advances in material screening and validation. Integrating standardized biological diagnostic frameworks into digital material selection processes may represent a key step toward future data-driven dental biomaterial development.

Therefore, the aim of this study was to propose a simplified in vitro diagnostic screening framework to evaluate the cytocompatibility of Mg–0.5Ca alloys enriched with increasing strontium content. By combining metabolic viability testing with fluorescence-based morphological assessment under standardized extract exposure, we provide a composition-dependent cytocompatibility profile relevant for preliminary selection of biodegradable metallic systems intended for dental and bone-related applications.

Unlike our previous comprehensive investigation focusing on advanced biological validation and in vivo assessment of Mg–Ca–Sr systems, the present study specifically proposes a simplified predictive in vitro diagnostic screening framework aimed at early-stage cytocompatibility profiling under standardized extract conditions. This approach provides emerging diagnostic insights relevant for preliminary composition selection in dental biomaterial development.

The study was designed as an ISO-standardized cytocompatibility screening step rather than a mechanistic ion-release investigation.

The novelty of the present study does not lie in demonstrating the isolated biological effects of strontium incorporation, which have been previously reported, but in the development of a standardized, ISO-compliant screening framework. This framework integrates material composition, extract preparation, physicochemical parameters such as pH, and biological response into a unified and reproducible workflow.

By using a controlled gradient of strontium content as a model system, the study aims to validate the sensitivity and applicability of this approach as a diagnostic tool for early-stage evaluation of dental materials.

## 2. Materials and Methods

### 2.1. Alloy Fabrication and Sample Preparation (Versiune Finală Curată)

The Mg–0.5Ca–xSr alloys (x = 0.5, 1, 1.5, 2, and 3 wt.%) were fabricated by conventional casting using a resistance-controlled laboratory furnace (REX C100, RKC Instruments Inc., Tokyo, Japan) under a continuous argon atmosphere to minimize oxidation during melting. High-purity elemental magnesium (≥99.9%), calcium, and strontium were weighed according to batch calculations and melted in ceramic crucibles at 720–750 °C. The molten alloys were maintained at this temperature range for approximately 15–20 min to ensure complete melting and chemical homogenization, followed by mechanical stirring under inert gas protection.

The liquid alloys were cast into preheated steel molds and allowed to solidify under argon shielding. Cooling was performed inside the furnace to minimize thermal gradients and residual stresses. The raw materials were procured via our collaboration with POKA (Bucharest, Romania) and were accompanied by supplier certificates of analysis to ensure traceability and reproducibility.

After solidification, the ingots were sectioned into standardized disc-shaped specimens. Prior to immersion and extract preparation, all samples were mechanically ground and polished using silicon carbide (SiC) abrasive papers with progressively finer grit sizes (P400, P800, P1200, and P2500) to ensure comparable surface conditions. The polished specimens were rinsed with distilled water and ethanol, dried under sterile conditions, and stored in aseptic containers until further testing.

The electrochemical and immersion corrosion testing procedures were performed following previously described methodologies reported in the literature [19].

The tests were performed using simulated body fluid (SBF) as electrolyte, prepared according to standard protocols.

**Figure 1:**
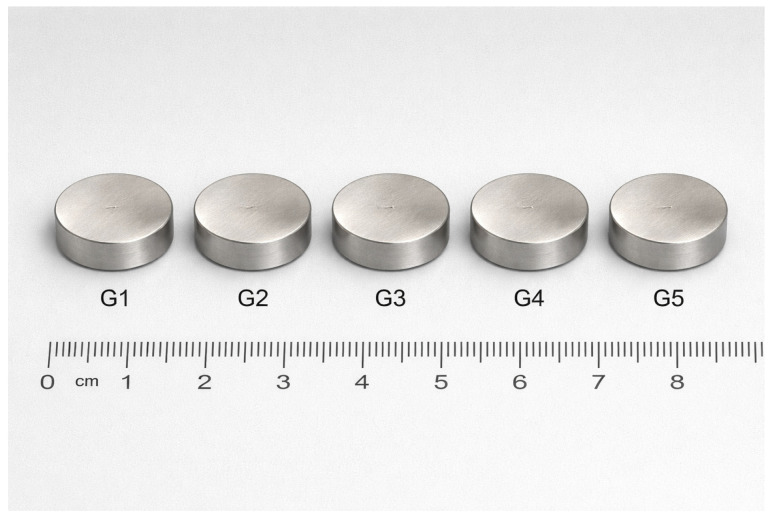
Representative macroscopic appearance of Mg–0.5Ca–xSr alloy specimens (G1–G5) after casting, sectioning, and surface preparation, prior to immersion for extract preparation according to ISO 10993-5:2009 [20] and ISO 10993-12:2012 [21] standards—based protocols. A metric scale is included to indicate specimen dimensions.

Representative macroscopic images of the prepared alloy specimens are presented in Figure 1, illustrating their geometry and surface integrity prior to biological evaluation.

### 2.2. Microstructural Characterization

Microstructural characterization of the Mg–0.5Ca–xSr alloys was performed using optical microscopy and scanning electron microscopy. Optical microscopy observations were carried out using a Leica DM microscope (Leica Microsystems GmbH, Wetzlar, Germany) to evaluate overall grain morphology and microstructural uniformity.

Detailed surface and microstructural features were examined by scanning electron microscopy (SEM) using a Tescan Vega II microscope (Tescan Orsay Holding, Brno, Czech Republic) operated in both high-vacuum and low-vacuum modes. Elemental surface composition was assessed by energy-dispersive X-ray spectroscopy (EDS) using an integrated EDS detector (Oxford Instruments, Oxford, UK) attached to the SEM system.

Phase identification of the alloys was conducted by X-ray diffraction (XRD) using a Bruker D8 Advance diffractometer (Bruker AXS GmbH, Karlsruhe, Germany) with Cu Kα radiation (λ = 1.5406 Å). Diffraction patterns were recorded over a 2θ range of 20–100°, with a step size of 0.02°, and the resulting spectra were analyzed using standard reference databases to identify crystalline phases and intermetallic compounds characteristic of Mg–Ca–Sr systems.

### 2.3. Electrochemical and Immersion Corrosion Testing

Electrochemical behavior was evaluated in simulated body fluid (SBF) and phosphate-buffered saline (PBS–NaCl) using a three-electrode configuration with a saturated calomel electrode as reference. Open-circuit potential (OCP), Tafel polarization curves, and cyclic polarization measurements were recorded. Immersion tests were performed for 72 h to monitor pH evolution and surface degradation. Post-corrosion morphologies were analyzed by SEM, EDS, and XRD.

Electrochemical measurements and immersion corrosion tests were performed in simulated body fluid (SBF) and phosphate-buffered saline (PBS–NaCl) solutions using a conventional three-electrode electrochemical cell. All experiments were conducted at a controlled temperature of 37 ± 1 °C in order to simulate physiological conditions. The electrolyte solutions were thermostatically maintained at this temperature throughout the testing period to ensure stable and reproducible corrosion behavior.

### 2.4. Preparation of Alloy Extracts

Prior to extract preparation, the alloy specimens were carefully cleaned to remove surface contaminants. Samples were rinsed with sterile deionized water, followed by disinfection in 70% (*v*/*v*) ethanol. Subsequently, the specimens were air-dried under sterile conditions.

Sterilization was performed by ultraviolet (UV) exposure for 30 min on each side, in accordance with the protocol previously applied for Mg–Ca–Sr alloy cytocompatibility testing. All drying and handling steps were carried out inside a Class II laminar flow cabinet (AURA 2000 M.A.C.), manufactured by BioAir S.p.A, Milan, Italy ensuring aseptic conditions prior to immersion in culture medium.

Extracts were prepared following ISO 10993-5 [20] and ISO 10993-12 [21] guidelines. Polished alloy discs were sterilized by UV exposure and immersed in complete MEM medium at a material-to-medium ratio of 0.1 g/mL for 48 h at 37 °C. Extracts were subsequently diluted to 10%, 20%, 40%, 60%, 80%, and 100% using fresh MEM.

No quantitative ion release measurements were performed, as the study was designed as an ISO-based cytocompatibility screening approach focusing on biological response to degradation extracts.

### 2.5. Cell Culture

Human osteoblast-like MG-63 cells were obtained from the American Type Culture Collection (ATCC, Manassas, VA, USA) and maintained according to supplier recommendations.

Cells were cultured in Minimal Essential Medium (MEM; Sigma-Aldrich, St. Louis, MO, USA) supplemented with 10% fetal bovine serum (FBS; Sigma-Aldrich, St. Louis, MO, USA) and 1% penicillin–streptomycin solution (Sigma-Aldrich, St. Louis, MO, USA). Cultures were maintained at 37 °C in a humidified atmosphere containing 5% CO_2_.

For routine passaging, cells were detached using Trypsin–EDTA solution (Sigma-Aldrich, St. Louis, MO, USA) and washed with phosphate-buffered saline (PBS; Sigma-Aldrich, St. Louis, MO, USA) prior to reseeding.

MG-63 cells were selected as a well-established osteoblast-like model widely used in biomaterials research due to their reproducibility, availability, and sensitivity to changes in the extracellular environment. Their use is also consistent with ISO 10993 [20,21] recommendations for initial in vitro cytocompatibility screening, making them suitable for standardized early-stage evaluation of biomaterials.

### 2.6. MTT Assay for Cell Viability

Cell viability was evaluated using the MTT assay (3-(4,5-dimethylthiazol-2-yl)-2,5-diphenyltetrazolium bromide; Sigma-Aldrich, St. Louis, MO, USA). After 24 h and 72 h of exposure to alloy extracts, the culture medium was removed and MTT solution was added to each well and incubated for 3 h at 37 °C.

Following incubation, the formed formazan crystals were dissolved in dimethyl sulfoxide (DMSO; Sigma-Aldrich, St. Louis, MO, USA). Absorbance was measured at 570 nm using a Spectramax Plus 384 Microplate Reader (Molecular Devices, San Jose, CA, USA), and cell viability was expressed as a percentage relative to untreated control cultures.

### 2.7. Fluorescence Imaging

After 72 h incubation with alloy extract dilutions, cells were washed with HBSS and stained with Calcein-AM (Sigma-Aldrich, St. Louis, MO, USA) for 30 min in the dark. Fluorescence images were acquired using an inverted fluorescence microscope (Leica DMIL LED, Germany).

### 2.8. Statistical Analysis

Quantitative data are presented as mean ± standard deviation (SD). Prior to statistical analysis, the assumptions required for one-way analysis of variance (ANOVA) were evaluated. Normality of data distribution was assessed using the Shapiro–Wilk test, while homogeneity of variances was verified using Levene’s test.

Comparisons among multiple groups were performed using one-way ANOVA, followed by Tukey’s post hoc test for pairwise comparisons when statistically significant differences were detected. A *p*-value < 0.05 was considered statistically significant.

All statistical analyses were carried out using GraphPad Prism software (version 9.0, GraphPad Software, San Diego, CA, USA).

### 2.9. Conducting In Vivo Analyses

For the in vivo tests, the use of animals was required, thereby imposing both scientific and ethical rigor. The study complied with the ethical directives presented in the Guide for the Care and Use of Laboratory Animals (Law 43/2014), the European legislation regarding animal use (EU/2010/63-CE86/609/EEC), and the animal experiments were approved by the Ethics Committee of the Faculty of Veterinary Medicine, “Ion Ionescu de la Brad” University of Life Sciences in Iași, Romania (Decision No. 1791/27/12/2024). No in vivo experiments were performed in this study.

### 2.10. Predictive In Vitro Diagnostic Screening Framework

To support early-stage evaluation of emerging dental biomaterials, a simplified in vitro diagnostic screening workflow was applied. Alloy extracts were prepared according to ISO 10993-5:2009 [20] and ISO 10993-12:2012 [21] standards recommendations, ensuring clinically relevant exposure conditions.

Cellular response was assessed through complementary quantitative and qualitative endpoints, including metabolic viability (MTT assay) and fluorescence-based live-cell morphology (Calcein-AM staining). This combined approach provides a predictive platform for identifying composition-dependent cytocompatibility trends prior to advanced biological validation.

## 3. Results

The in vitro findings were interpreted within a predictive diagnostic screening perspective, aiming to generate a composition-dependent cytocompatibility profile across increasing Sr concentrations. The combined endpoints, including metabolic viability (MTT assay) and fluorescence-based live-cell morphology (Calcein-AM), were used to identify consistent early biological response trends under standardized extract exposure conditions. This approach supports preliminary composition ranking prior to advanced mechanistic or in vivo validation.

### 3.1. Characterisation of Mg–Ca–Sr Alloy Series and Extract Preparation

The series of Mg–0.5Ca–xSr biodegradable alloys investigated in this study comprised five compositions with graded strontium content (x = 0.5, 1.0, 1.5, 2.0 and 3.0 wt.%), labelled G1–G5, respectively. The alloy codes and nominal compositions are summarised in Table 1. These materials were elaborated under controlled atmosphere by melting high-purity elements, followed by casting into metallic moulds and subsequent sectioning and surface preparation for degradation and biological testing.

Degradation extracts were obtained by immersing polished alloy specimens in complete MEM culture medium for 48 h at 37 °C, at a fixed surface-area-to-volume ratio, according to ISO 10993-5:2009 [20] and ISO 10993-12:2012 [21] standards guidelines. The resulting stock extracts (100%) were clear, particle-free solutions which were subsequently diluted with fresh medium to obtain a series of working concentrations (10–100%). The corresponding extract and medium fractions used in the experiments are detailed in Table 2.

**Figure 2:**
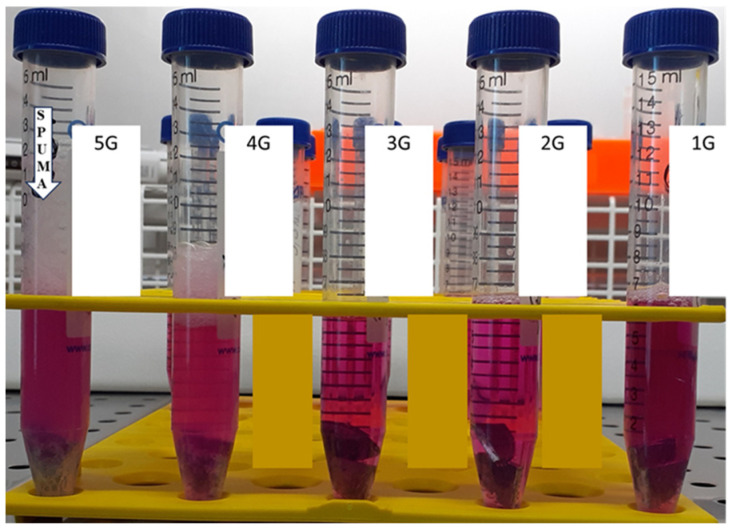
Macroscopic aspect of Mg–0.5Ca–xSr alloy samples and the corresponding degradation extracts obtained after 48 h immersion in complete MEM medium at 37 °C.

The macroscopic appearance of the alloy specimens immersed in complete MEM medium and of the corresponding degradation extracts after 48 h is illustrated in Figure 1. No visible precipitation or turbidity was observed, and all extracts remained optically clear, suggesting the absence of particulate detachment or gross instability under the selected extraction conditions (Figure 2).

### 3.2. MG-63 Cell Viability Following Exposure to Alloy Extracts (MTT Assay)

The metabolic activity of osteoblast-like MG-63 cells exposed to Mg–0.5Ca–xSr alloy extracts was assessed after 24 h and 72 h of incubation. Across all compositions, none of the tested extract conditions induced cytotoxicity, while several Sr-containing alloys demonstrated enhanced metabolic activity at 72 h.

**Figure 3:**
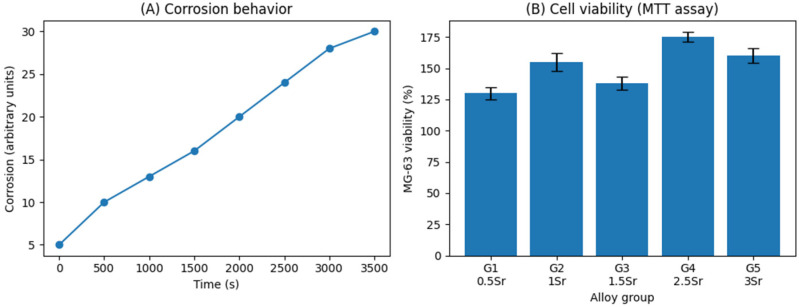
Evaluation of Mg–Ca–Sr alloys. (**A**) Corrosion behavior over time. (**B**) MG-63 cell viability after exposure to alloy extracts, assessed by MTT assay. Data are presented as mean ± SD.

The detailed MTT dataset has been previously reported; here, viability outcomes are summarized to support correlation with extract alkalinization behaviour (Figure 3).

To provide a physicochemical context for these findings, immersion testing revealed a progressive alkalinization of the degradation medium. In particular, the pH of SBF increased from the physiological baseline (~7.4) toward values approaching 10 over 72 h for the highest Sr-containing alloy (G5), consistent with magnesium dissolution and hydroxide generation. The highest Sr-containing alloy (G5) exhibited the most pronounced alkalinization trend, consistent with magnesium dissolution and hydroxide generation. However, exact pH values of the cell culture extracts were not quantitatively determined in this study, and the observed effect is interpreted based on known corrosion behavior.

**Figure 4:**
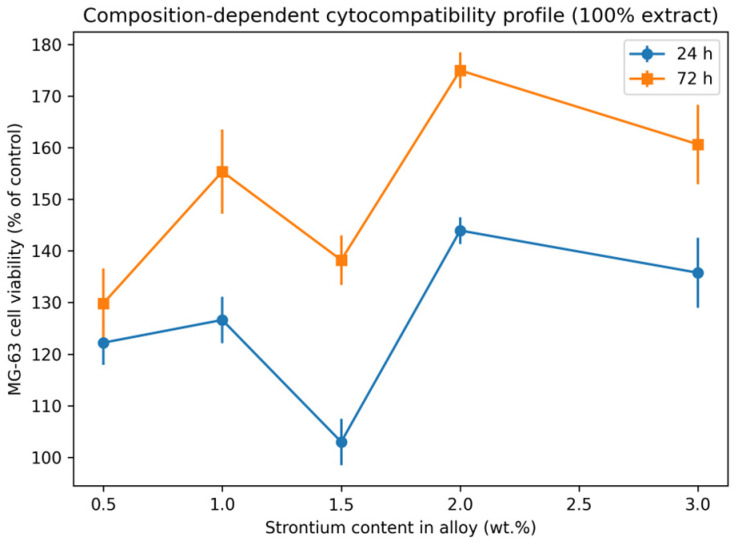
Mg alloys enriched with increasing strontium content (0–2 wt.%); MG-63 metabolic viability was assessed by MTT assay after 24 and 72 h of exposure to standardized degradation extracts. Results are expressed as a percentage relative to untreated control cells. The profile highlights Sr-dependent trends suitable for preliminary composition ranking.

A combined representation of viability outcomes and extract alkalinization behaviour is provided in Figure 4, illustrating that elevated MG-63 metabolic activity can occur within a biologically tolerable alkaline window, supporting the role of Sr incorporation in modulating early cellular response.

Across the tested range, the Sr-containing alloys exhibited a more favorable cytocompatibility profile than the Sr-free control, suggesting that Sr incorporation may modulate extract-driven cellular response in a concentration-dependent manner under these standardized conditions.

To further highlight the composition-dependent response, viability was plotted as a function of strontium content, generating a predictive cytocompatibility profile suitable for preliminary alloy ranking (Figure 4).

This representation allows a clearer diagnostic-style visualization of Sr-driven trends across both exposure times. The representation of viability as a function of strontium content enables visualization of a composition-dependent cytocompatibility profile. Across both exposure times, Sr-containing alloys demonstrated a more favorable metabolic response compared with lower-Sr compositions, with the highest stimulatory effect observed at 2 wt.% Sr after 72 h. This diagnostic-style profiling supports preliminary alloy ranking prior to advanced mechanistic validation.

### 3.3. Morphological Response of MG-63 Cells to Alloy Extracts

The qualitative morphology of MG-63 cells after 72 h exposure to undiluted (100%) alloy extracts was examined by fluorescence microscopy following Calcein-AM staining. Across all alloy groups, cells exhibited an adherent, well-spread osteoblastic phenotype, with polygonal cell bodies and numerous cytoplasmic extensions. No evidence of cell rounding, membrane blebbing, or fragmentation was observed, which corroborates the non-cytotoxic behaviour indicated by the MTT data.

**Figure 5:**
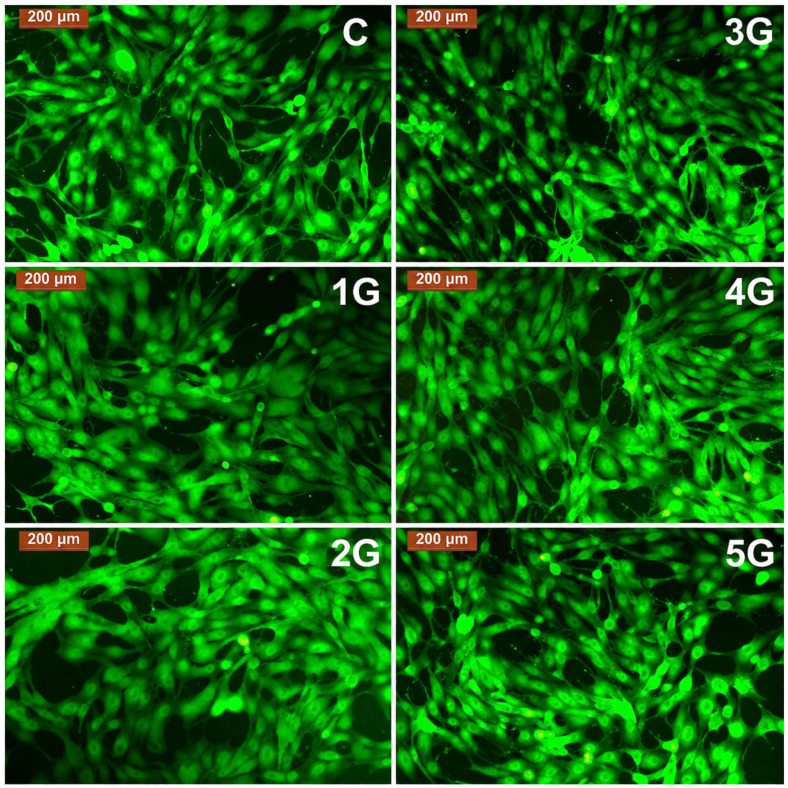
Representative fluorescence microscopy images illustrating the morphology of osteoblast-like MG-63 cells following 72 h exposure to undiluted (100%) degradation extracts from the Mg–Ca–Sr alloy series (G1–G5) and control cultures (C). Calcein-AM staining reveals well-spread osteoblastic morphology and preserved membrane integrity. Scale bar: 200 µm.

Representative fluorescence micrographs for each alloy group (G1–G5) and for control cultures are shown in Figure 2. In all conditions, MG-63 cells formed continuous monolayers with preserved cell–cell contacts and homogeneous Calcein-derived fluorescence, indicating intact membrane integrity and esterase activity. Samples corresponding to higher Sr contents (G4–G5) displayed particularly dense cell layers with intense fluorescence and extensive intercellular networking, consistent with the increased proliferative indices recorded at 72 h (Figure 5).

## 4. Discussion

The present study evaluated the biological response of MG-63 osteoblast-like cells to degradation extracts derived from a compositional gradient of Mg–0.5Ca–xSr alloys. The results demonstrated clear cytocompatibility across all groups, with enhanced viability and robust morphological behavior, particularly associated with higher Sr contents. These outcomes are consistent with trends reported in the biomaterials literature of the last five years, which increasingly highlight the strong osteogenic potential of strontium-modified magnesium systems [22].

Recent studies (2020–2024) have emphasized that controlled Mg degradation and the consequent ionic release can stimulate osteoblastic activity when local physicochemical changes remain within physiological boundaries. The clear appearance and stable pH of extracts obtained in this study align with observations that moderate Mg^2+^ release supports early adhesion and proliferative responses through pathways such as integrin signaling and modulation of intracellular calcium balance. Similarly, Ca^2+^ ions contribute to osteoblast differentiation via activation of calcium-sensing receptors, a mechanism widely confirmed in recent cell–material interaction research [23,24,25].

In comparison with existing methodologies, most studies evaluating dental biomaterials focus on isolated parameters, such as cytotoxicity assays, ion release profiles, or osteogenic marker expression. These approaches, although valuable, are often performed independently and lack integration within a standardized workflow.

In contrast, the framework proposed in the present study combines ISO 10993-compliant [20,21] extract preparation with physicochemical characterization, including pH assessment, and biological evaluation using cell viability and morphology. This integrated approach allows for a more comprehensive and reproducible assessment of material behavior in early-stage testing.

The added value of this methodology lies in its potential to function as a rapid and standardized screening tool, facilitating the selection and optimization of biomaterials before advancing to more complex and resource-intensive experimental models.

It is important to emphasize that the biological behavior observed in the present study should be interpreted in the context of previously reported materials characterization data for the same Mg–0.5Ca–xSr alloy series. Earlier investigations demonstrated that controlled Sr additions refine the alloy microstructure, influence corrosion kinetics, and support favorable in vivo tissue responses [17,18]. By isolating the effect of degradation extracts in the current work, we aimed to decouple ionic-mediated cellular responses from surface-related phenomena, thereby providing clearer insight into how Mg^2+^, Ca^2+^, and Sr^2+^ release modulates osteoblast-like cell behavior.

This extract-based approach is widely accepted for preliminary biological screening of biodegradable metals, as it minimizes confounding effects related to hydrogen evolution, surface roughness, and transient corrosion layers, which can obscure early cellular responses in direct-contact systems. Within this framework, the enhanced metabolic activity and robust morphology observed, particularly for higher Sr-containing alloys, support the cytocompatibility of Mg–Ca–Sr systems and their potential to create a biologically supportive ionic microenvironment.

The pronounced viability increases observed for alloys with higher Sr content (G3–G5) are also consistent with numerous in vitro experiments demonstrating that Sr^2+^ enhances osteoblast proliferation, collagen production, and ALP activity. In recent biomaterials engineering publications, Sr-containing magnesium alloys, glasses, and ceramics have shown superior performance in supporting bone-related cells [26], and this effect is attributed to Sr’s ability to simultaneously stimulate osteoblastogenesis and suppress osteoclast activity. The viability ranges recorded at 72 h in this study (often surpassing 130–140% of control) are well aligned with such reports, suggesting that Sr-enriched Mg-based alloys may offer biologically favorable ionic environments for osteoregenerative applications [27].

MTT results further reflect time-dependent adaptation of MG-63 cells to the extract environment. Similar findings in recent biodegradation studies describe that osteoblast-like cells often experience minor metabolic fluctuations within the first 24 h but subsequently exhibit enhanced proliferation once ionic concentrations stabilize. The overall increase in viability between 24 h and 72 h in this study supports this adaptive behavior, emphasizing the importance of evaluating both short- and mid-term cellular responses [28,29].

Fluorescence microscopy confirmed the favorable cellular behavior suggested by the MTT assay. Cells exhibited polygonal spreading, extensive cytoplasmic projections, and intact membrane integrity under all extract conditions, reflecting normal osteoblastic morphology. These features are supported by recent morphological analyses in the literature, where Mg-based materials that maintain cytoskeletal organization and promote lamellipodia formation are considered highly cytocompatible. The increased cell density and fluorescence intensity observed in G4 and G5 groups further reinforce the beneficial influence of Sr release [30,31].

Nevertheless, while the results clearly support the cytocompatibility of these alloys, several aspects warrant further attention. Current high-impact literature increasingly calls for integrating molecular markers—such as RUNX2, COL1A1, OPG/RANKL ratios, and osteocalcin expression—to more comprehensively characterize osteogenic activity induced by Mg- and Sr-containing biomaterials. Additionally, although the extract-based method provides valuable insight into ionic effects, direct-contact studies incorporating surface topography and corrosion layer morphology would complement these findings and align with the methodological standards of recent in vitro evaluations [32,33].

Furthermore, contemporary research warns that excessive Sr additions may accelerate galvanic corrosion by promoting noble intermetallic formation. While the present study did not observe cytotoxic effects, future work should assess long-term degradation kinetics and cumulative ion release to ensure compositional optimization from both biological and corrosion perspectives [34,35,36]. Although the present results consistently indicate enhanced cytocompatibility for Sr-containing Mg–Ca–Sr alloys, it should be emphasized that quantitative measurements of ion release were not performed in this study. As a result, the proposed contribution of Sr^2+^ ions to the observed cellular responses is based on indirect evidence derived from alloy composition, extract preparation protocols, and previously reported degradation behavior of similar systems.

It should be emphasized that quantitative measurements of Mg^2+^, Ca^2+^, and Sr^2+^ ion release were not performed in the present study. This is consistent with the primary objective of the work, which was to establish a standardized biological screening framework rather than to provide a detailed physicochemical analysis of degradation kinetics.

In extract-based cytocompatibility studies, biological responses are often interpreted as the combined effect of ionic species and changes in the local microenvironment, including pH variations. Therefore, the present approach focuses on the integrated cellular response to degradation extracts, rather than on isolated ion concentration measurements.

Nevertheless, quantitative ion release analysis using techniques such as ICP-OES or ICP-MS would represent an important extension of the present work and is planned for future investigations to further correlate compositional parameters with biological outcomes.

In extract-based cytocompatibility studies, quantitative determination of released metal ions using techniques such as inductively coupled plasma optical emission spectroscopy (ICP-OES) or atomic absorption spectroscopy is recognized as an important tool for establishing a direct correlation between degradation kinetics and biological effects. The absence of such analyses represents a limitation of the present work and should be addressed in future investigations to more precisely elucidate the dose-dependent role of Sr^2+^ ions in modulating osteoblast-like cell behavior.

While the stimulatory effect of strontium on osteoblast-like cells has been extensively described in the literature, the present study extends these findings by embedding them within a structured screening methodology. Rather than focusing on isolated biological outcomes, our approach emphasizes the integration of physicochemical and biological parameters into a coherent framework designed for reproducibility and translational applicability.

Overall, the findings presented here are in strong agreement with the trajectory of recent biomaterials literature, supporting Mg–Ca–Sr alloys as attractive candidates for biodegradable implant applications. Their ability to promote osteoblast viability while maintaining cytocompatible degradation profiles suggests significant translational potential in bone tissue engineering. The positive cellular outcomes observed across all extract concentrations provide a strong foundation for continued optimization and future in vivo validation.

The reduced cell viability observed at intermediate extract concentrations may reflect a non-linear biological response, potentially influenced by a balance between beneficial ionic stimulation and adverse microenvironmental changes, including osmotic effects and local alkalinization.

The present findings emphasize the importance of predictive diagnostic screening assays in the early development of biodegradable dental biomaterials. While comprehensive in vivo validation remains essential, simplified in vitro diagnostic platforms enable rapid identification of biologically favorable compositions, supporting efficient translational decision-making.

While the present study demonstrates favorable short-term cytocompatibility of Mg–Ca–Sr alloy degradation extracts, it is important to consider the potential implications of long-term degradation behavior and cumulative ion release. Over-extended implantation periods, progressive magnesium matrix dissolution may lead to sustained release of Mg^2+^, Ca^2+^, and Sr^2+^ ions, potentially altering the local chemical environment and cellular responses in a time-dependent manner.

In addition, higher strontium contents may influence micro-galvanic interactions within the alloy microstructure, which could affect long-term corrosion kinetics and ion release profiles. Although such effects were not directly assessed in the present extract-based study, they represent relevant factors for the long-term biological performance of Mg–Ca–Sr systems.

In vivo, the degradation rate of magnesium-based alloys is influenced by multiple factors, including alloy composition, local tissue environment, and implant geometry, and may range from several weeks to months. The present study does not address long-term degradation kinetics, as it is focused on early-stage in vitro screening.

Future investigations combining prolonged immersion experiments, quantitative ion release measurements, and long-term in vitro or in vivo models will be essential to more comprehensively evaluate cumulative effects and to optimize alloy composition for sustained clinical performance.

### Limitations of the Study

This study was designed as an early-stage diagnostic screening approach and therefore focused on extract-based cytocompatibility endpoints, including metabolic viability and qualitative cell morphology. While this methodology provides valuable preliminary insight into the biological tolerance of Mg–Ca–Sr degradation products, several limitations should be acknowledged.

First, the present work did not include direct quantification of released ionic species (Mg^2+^, Ca^2+^, and particularly Sr^2+^) using analytical techniques such as ICP-OES or ICP-MS. As a result, composition-associated biological trends cannot be interpreted as confirmed dose–response relationships. Future investigations combining ion concentration profiling with biological outcomes will be essential to strengthen predictive modeling.

Second, osteogenic differentiation endpoints were not evaluated. Although MG-63 viability remained preserved or enhanced under several extract conditions, functional markers of osteoblast maturation (e.g., ALP activity, RUNX2, COL1A1, or OCN expression) were beyond the scope of this screening study. Such differentiation-related readouts will be required to determine whether metabolic stimulation translates into true osteogenic potential.

Third, only one osteoblast-like cell line (MG-63) was employed. While widely used for preliminary cytocompatibility assessment, MG-63 cells represent an osteosarcoma-derived model and may not fully reflect primary human osteoblast behavior. Additional validation using clinically relevant cell types, such as primary osteoblasts or mesenchymal stromal cells, is warranted.

While MG-63 cells provide a reproducible and standardized model for early screening, further validation using primary human osteoblasts or mesenchymal stem cells would be required to fully assess clinical relevance.

Primary osteoblasts and mesenchymal stem cells (MSCs) differ significantly in their biological relevance and experimental behavior. Primary osteoblasts are terminally differentiated cells that directly reflect bone-forming activity and are therefore highly representative of mature bone tissue function. However, they are characterized by donor-dependent variability, limited lifespan, and reduced reproducibility.

In contrast, MSCs represent a multipotent progenitor population capable of differentiating into osteoblasts under appropriate conditions. They are particularly valuable for studying osteogenic differentiation potential and regenerative processes, but their behavior is strongly influenced by culture conditions and differentiation protocols.

Compared with these models, MG-63 cells offer a stable and reproducible system suitable for standardized cytocompatibility screening, although they do not fully replicate the complexity of primary human bone cells.

Moreover, the evaluation was limited to extract-based exposure, which captures primarily ionic and soluble degradation effects but does not account for direct cell–material interactions, surface topography, corrosion layer formation, or adhesion-dependent responses. Direct-contact assays would provide complementary information regarding cytoskeletal organization and surface-mediated bioactivity.

Additionally, although magnesium degradation is known to induce local alkalinization, pH variations in the extracts were not quantitatively measured in this study. As a result, potential pH-mediated effects on MG-63 cell behavior could not be distinguished from ion-specific contributions. Future studies should integrate real-time pH monitoring to better correlate physicochemical changes with cellular responses.

Finally, the extract preparation represents a short-term degradation snapshot (48–72 h). Magnesium alloys undergo continuous corrosion, and the physicochemical environment may evolve substantially over longer periods. Long-term degradation studies integrating corrosion kinetics, hydrogen evolution, and mechanical integrity are necessary before clinical translation.

Overall, future extensions of this diagnostic framework should integrate ion release quantification, osteogenic differentiation markers, direct-contact models, and longer-term degradation behavior to refine the translational relevance of Mg–Ca–Sr biodegradable alloys.

## 5. Conclusions

Within a simplified in vitro diagnostic screening framework, Sr-enriched Mg–0.5Ca alloys exhibited a more favorable cytocompatibility and morphology profile in MG-63 cells compared with lower-Sr compositions under standardized extract exposure. These findings provide emerging, composition-dependent screening insights that may support preliminary selection of biodegradable Mg-based systems for dental and bone-related applications. The proposed framework can serve as a practical early-stage platform that can be expanded in future work through extract chemistry correlation and osteogenic differentiation readouts to enhance predictive value. Despite its limitations, the proposed approach fulfills its role as an early-stage diagnostic screening tool, allowing preliminary composition-dependent ranking prior to more advanced mechanistic or in vivo investigations.

Future studies integrating ion-release quantification and osteogenic differentiation markers are required before clinical translation.

## Figures and Tables

**Table 1:** Alloy codes and nominal compositions of the Mg–0.5Ca–xSr series.

Alloy Code	Nominal Composition	Sr Content (wt.%)
G1	Mg–0.5Ca–0.5Sr	0.5
G2	Mg–0.5Ca–1Sr	1.0
G3	Mg–0.5Ca–1.5Sr	1.5
G4	Mg–0.5Ca–2Sr	2.0
G5	Mg–0.5Ca–3Sr	3.0

**Table 2:** Extract dilutions used for co-incubation with MG-63 cells.

Extract Designation	Extract (%)	Fresh Medium (%)
Extract 10%	10	90
Extract 20%	20	80
Extract 40%	40	60
Extract 60%	60	40
Extract 80%	80	20
Extract 100%	100	0

## Data Availability

Data are contained within the article.
